# Severe Progressive Multifocal Leukoencephalopathy (PML) and Spontaneous Immune Reconstitution Inflammatory Syndrome (IRIS) in an Immunocompetent Patient

**DOI:** 10.3389/fimmu.2019.01188

**Published:** 2019-05-28

**Authors:** Lea Krey, Peter Raab, Romilda Sherzay, Georg Berding, Matthias Stoll, Martin Stangel, Florian Wegner

**Affiliations:** ^1^Department of Neurology, Hannover Medical School, Hanover, Germany; ^2^Hannover Medical School, Institute of Neuroradiology, Hanover, Germany; ^3^Department of Nuclear Medicine, Hannover Medical School, Hanover, Germany; ^4^Clinic for Immunology and Rheumatology, Unit for Infectious Diseases, Hannover Medical School, Hanover, Germany

**Keywords:** JC-virus, immunocompetence, progressive multifocal leukoencephalopathy (PML), spontaneous immune reconstitution inflammatory syndrome (IRIS), corticosteroid

## Abstract

**Background:** Progressive multifocal leukoencephalopathy (PML) is an opportunistic infection with JC-virus (JCV), a papova-virus, affecting mostly oligodendrocytes and the white matter of the central nervous system. Progressive Multifocal Leukoencephalopathy (PML) almost exclusively occurs in immunocompromised patients based on different underlying conditions of severe cellular immunodeficiency such as HIV/AIDS, secondary to neoplastic and autoimmune diseases, or during immunosuppressive therapy.

**Case presentation:** We present the case of an otherwise healthy and immunocompetent patient without immunosuppressive therapy who was admitted with hemianopsia to the right side, sensory aphasia and changes of behavior. Magnet resonance imaging (MRI) and laboratory testing confirmed the diagnosis of PML, although functional tests did not show any evidence for cellular immunodeficiency. Extensive immunological tests did not reveal an apparent immunodeficiency. During symptomatic therapy the patient developed seizures which were assumed to be caused by a spontaneous immune reconstitution inflammatory syndrome (IRIS) demonstrated by MRI. We added a high dose of intravenous corticosteroids to the antiepileptic treatment and seizures ended shortly thereafter. However, the impairments of vision, behavior and language persisted.

**Conclusions:** Our case report highlights that an apparently immunocompetent patient can develop PML and IRIS spontaneously. Therefore, MRI should be applied immediately whenever a rapid progression of PML symptoms occurs as treatment of IRIS with corticosteroids can result in a marked clinical improvement.

## Introduction

Progressive Multifocal Leukoencephalopathy (PML) is an opportunistic infection of white and gray matter cells caused by JCV-reactivation within the brain almost exclusively occurring in immunocompromised patients. Hence, PML has been explained as an opportunistic infection secondary to underlying predominantly cellular forms of immunodeficiencies (e.g., HIV/AIDS, neoplastic and autoimmune diseases or immunosuppressive treatments such as rituximab, natalizumab, and others) (1, 2). JCV latency in healthy individuals is common with 50–90% of the population being positive for JCV-antibodies (2). The incidence of PML is comparably low (4.4 of 100,000), as a severe impairment of specific parts of the immune system (especially CD4+-T-lymphocytes) is necessary for intracerebral virus reactivation (2). The prognosis of PML is often poor. There is no specific therapy with proven efficacy except for a sufficient treatment of immunodeficiency (e.g., antiviral treatment of HIV) (3). But on the other hand also the reconstitution of the deficient cellular immune system can lead to an excessive immune activation against the JCV called IRIS (4, 5). A PML-IRIS treatment with high doses of corticosteroids is recommended (5, 6). Nevertheless, the course of PML frequently remains unfavorable leading to prolonged neurological impairment or death of affected patients.

We present a case of PML in a patient with no detectable immunodeficiency at time of PML diagnosis. As far as neuroimaging by cMRI suggested a subsequent development of an IRIS, a corticosteroid-treatment was initiated resulting in a marked benefit of the PML.

## Case Presentation

**Figure 1:**
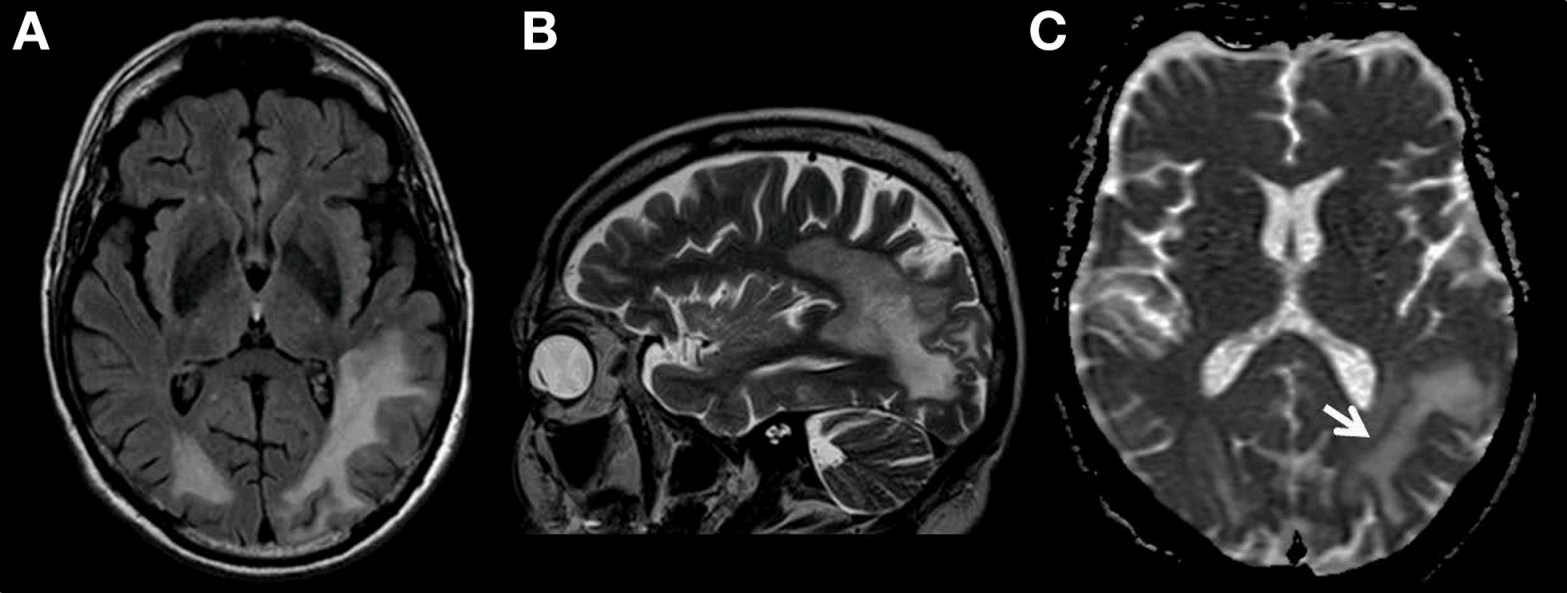
MR examination showing typical findings of PML with white matter FLAIR- **(A)** and T2 weighted- **(B)** hyperintensities without mass effect. T2-signal elevations **(B)** are in part spot-like and resemble the milky-way-appearance typical for PML. The area affected by the JC-virus infection does have a typical rim with reduced ADC values (**C**, white arrow) indicating the inflammatory border zone with acute demyelination combined with swelling of astrocytes and oligodendrocytes.

A 68-year-old male patient was transferred to our neurological department after he had been admitted to another hospital twice before. The first admission had taken place 3 months before in July 2018 due to visual impairments (hemianopsia to the right) and uncommon behavior. The MRI had shown a T2-weighted (T2w)-FLAIR hyperintensity in the left temporooccipital lobe that had been interpreted as an ischemic stroke. The results of cardiovascular investigations had been normal and the patient had been discharged. In August 2018 he had been admitted again with a progression of symptoms consisting of severe sensory aphasia, psychomotoric deficits and progressive visual impairments. The MRI had displayed a massive enlargement of parietooccipital lesions in both hemispheres predominantly affecting the left white matter (Figure 1). The polymerase chain reaction (PCR) for JCV in the cerebrospinal fluid (CSF) had been positive whereas routine analysis of the CSF had shown normal results (cell count and distribution, lactate, protein). PCR for Varizella zoster virus had been negative. Serologic tests for Borrelia burgdorferi, Treponema pallidum, Herpes simplex virus, measles, Varizella zoster virus, HIV and hepatitis B and C had also been negative.

The patient was then transferred to our department for further diagnostics. His general physician and relatives revealed that the medical history contained no record of frequent or severe infections. He had never received any kind of immunosuppressive treatment. In 1999 the patient had suffered from a myocardial infarction. The medical history was unremarkable apart from arterial hypertension and a helicobacter-positive gastritis. In 2017 he had got a viral infection with mild influenza-like-symptoms. Neither the patient nor his family knew of any recent vaccinations.

Upon admission to our neurologic department he had developed a right sided homonymous hemianopsia, severe sensory aphasia and disorientation. Our CSF analysis showed a pleocytosis (22 cells/μl) and an elevated protein concentration (0.72 g/l). The cell-distribution was mostly lymphocytic, but monocytes and granulocytes were found as well. CSF specific oligoclonal bands (type 2) were present and there was an intrathecal production of IgG and IgM. The JCV-PCR (1E3 c/ml) from CSF and the JCV-antibody-index (24.000 AU/ml serum) were positive. All additional tests were negative (HSV, VZV, and Ebstein-barr-virus (EBV) in the CSF by PCR, as well as tickborn encephalitis (TBE), enterovirus, and cytomegalovirus serologically; CSF-antibody-indices for HSV, EBV, VZV and CMV). All investigated autoimmune encephalitis antibodies (anti-NMDAR, -AMPAR1/2, -CASPR2, -LGI1, -GABAR-b1/b2, -Yu, -Hu, -Ri, -Amphiphysin, -CV2/CRMP-5, -Ma1/2, -GAD, -SOX1, -Tr(DNER), -Zic4) were negative in serum and CSF.

**Figure 2:**
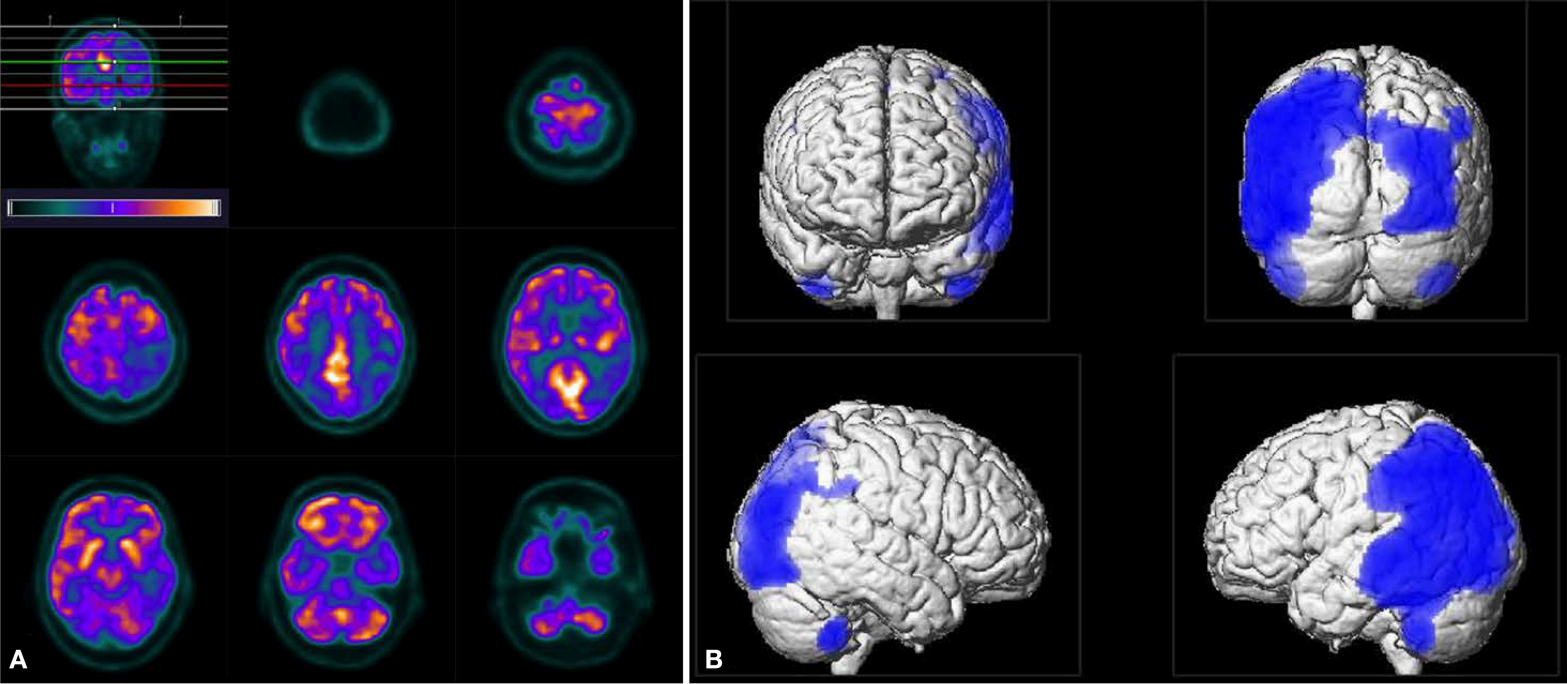
Tomograms of ^18^F-FDG uptake **(A)** as well as the result of SPM analysis (**B**, using an uncorrected *p* < 0.001 for statistical inferences) revealed distinct bilateral hypo-metabolism pronounced on the left side in parietotemporal and occipital areas. Note, unlike in tomograms the left side appears left in the SPM images.

Further investigations concerning an immunodeficiency were inconspicuous. An HIV-test was negative. The blood tests including peripheral blood cell count and inflammatory parameters showed mild eosinophilia (10,3%), elevated rheumatoid factor (60.5 IU/ml), Cardiolipin-IgM-antibodies (14 MPL-U/ml), and antinuclear antibodies (ANA, 1:320) of unspecific speckled pattern in the immunofluorescence and without specificity against ds-DNA or extractable nuclear antigens. An abdominal sonography showed a splenomegaly and prominent lymph nodes but no sign of neoplasia. We performed a whole-body PET/CT (including the brain) with 296 MBq ^18^F-Fluorodeoxyglucose (^18^F-FDG) 60 min p.i. to search for a malignancy or an inflammatory disease. The PET scan showed prominent mediastinal lymph nodes with moderately high metabolism, however, it did not reveal any profound hypermetabolic lesions. ^18^F-FDG uptake in the brain was further evaluated using statistical parametric mapping (SPM) for comparison with a reference group. The cerebral glucose metabolism was lowered bilaterally in parietotemporal and occipital areas with a clear predominance on the left side. The metabolism in the frontal cortex, basal ganglia, and cerebellar regions appeared to be normal (Figure 2).

A biopsy of the prominent mediastinal lymph nodes identified in the PET investigation showed no signs of malignancy or granuloma. Additionally ACE and the soluble IL-2-receptor in the blood were normal, so sarcoidosis seemed unlikely. The HLA-DR-expression and phenotyping of lymphocytes showed normal results [amount and distribution of CD3+, CD4+, CD8+, B-cells (CD20+), and natural killer cells (CD16+)]. In addition to extensive phenotyping of peripheral blood mononuclear cells (PBMC) even the functional analyses by proliferation assays of PBMC by plant lectins (PHA, Con A, PWM), tuberculin (PPD) and anti-CD3, spontaneous and antibody-dependent NK-cell activity (ADCC) were within normal range.

**Figure 3:**
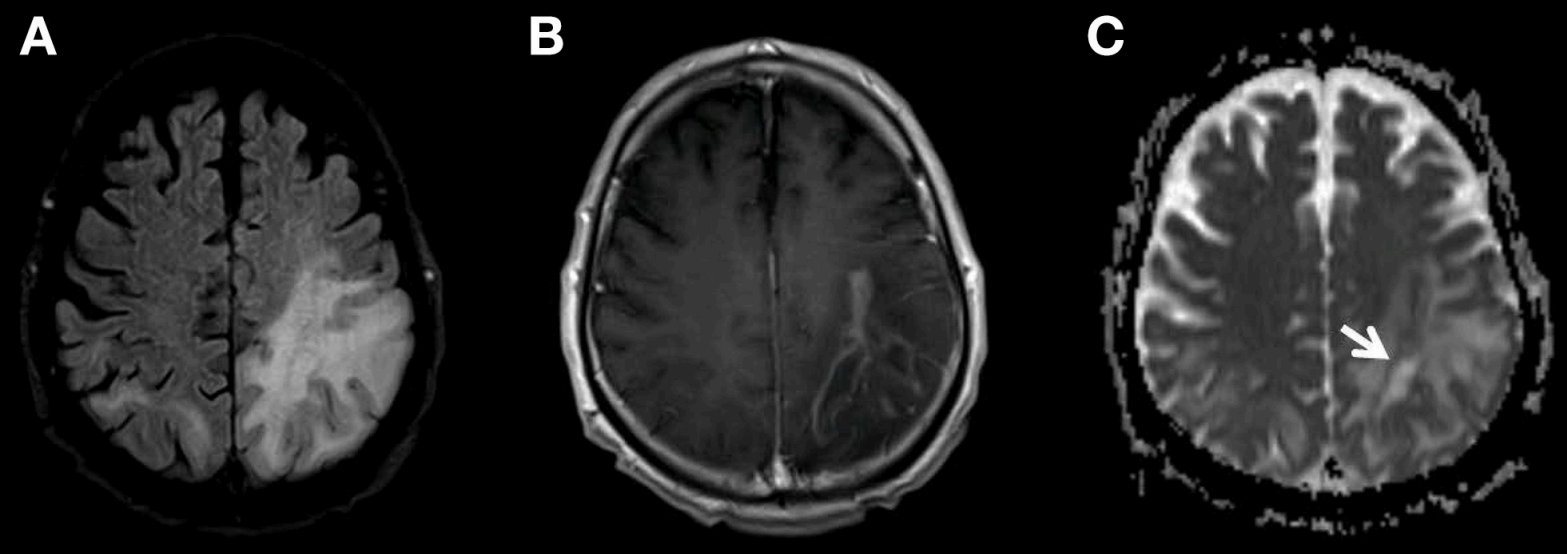
MR examination showing the progress of the PML-related FLAIR-hyperintensities **(A)**. The new multifocal and strong contrast enhancement **(B)** indicates the PML-IRIS. The zone of active inflammation and demyelination is again indicated also by the low ADC-rim surrounding the area affected by the JC-virus (**C**, white arrow).

During inpatient treatment the patient developed a cough and had impaired breathing. The tracheal exudate was positive for HSV using PCR so we started a therapy with intravenous aciclovir which improved the respiratory symptoms markedly. In September 2018 a progression of the patient's symptoms with focal and generalized seizures occurred. We started a medication with levetiracetam and had to add valproate and lacosamide to the antiepileptic treatment due to recurring seizures. The MRI showed a further expansion of the lesions with a new peripheral contrast enhancement (Figure 3) as a sign of a PML-related immune reconstitution inflammatory syndrome (PML-IRIS). We treated the patient with a high dose of corticosteroids (1 g/d methylprednisolone for 5 days intravenously). After this therapy the seizures decreased and the anticonvulsive treatment could be reduced to levetiracetam and valproate. Finally, we applied a treatment with mirtazapine to our patient, which is able to block the specific serotonin-receptors used by JCV, since this was beneficial in a previous report of a PML in an immunocompetent patient (7). Mefloquine, another drug with potentially antiviral effects against JCV, was not included to the treatment as seizures are a strict contraindication (7).

After nearly 2 months the patient was transferred to a rehabilitation facility. Visual impairments, disorientation and a sensory aphasia were still persistent. During the following weeks the patient developed severe dysphagia until oral intake of food and fluids was no longer possible. The patient died 2 months after the transfer to a nursery home due to a respiratory infection and pulmonary insufficiency.

## Discussion and Conclusions

The presented case shows evidence for the rare development of severe PML and spontaneous IRIS in a patient with no apparent immunodeficiency. A neoplastic disease could not be detected by abdominal ultrasound, thoracic X-ray and a whole-body ^18^F-FDG-PET/CT scan. HIV-results were repeatedly negative. A chronic autoimmune disease was also not detected and a biopsy of mediastinal lymph nodes showed no sign of sarcoidosis. Blood tests revealed a normal distribution and function of PBMC, especially the amount of CD4+-cells was normal. However, we cannot exclude a stage of transient immunodeficiency before PML developed, e.g., secondary to an intercurrent viral infection which had presented “flu-like” and clinically self-limiting in 2017. However, an episode of symptomatic tracheobronchitis with HSV-reactivation shown in the BAL in 09/2018 might indicate persisting immunodefiency, although we did not find further evidence for a capable immunological impairment at the same time. Hence, a hypothetical explanation of the course of PML in the presented case may be a (a) transient cellular immunodeficiency potentially due to the suspected viral infection in 2017 as the predisposition for development of PML and JCV-reactivation; (b) clinical development of PML as a demasking IRIS since summer 2018 during spontaneous immunoreconstitution; (c) further progression of PML-IRIS after antiviral treatment of HSV-tracheobronchitis in 09/2018 and (d) clinical improvement by corticosteroid-pulse subsequently. The HSV reactivation in the respiratory tract would be in accordance to this hypothetical course because it is known to be caused by immunodeficiency and as well by IRIS (8, 9).

Few comparable cases with PML in apparently immunocompetent patients have been published (10–15) but a spontaneous PML-IRIS has not been demonstrated in those cases. In one case the development of a PML-IRIS was suggested but no evidence (MRI) of IRIS was demonstrated (16). Our case shows MRI-evidence of a new onset PML-IRIS that occurred spontaneously and caused rapidly progressive neurological symptoms. Treatment with high doses of corticosteroids led to marked clinical improvement. Nevertheless, severe neurological impairments persisted due to the PML. Hypometabolism in ^18^F-FDG PET/CT might indicate mild infiltration by inflammatory cells (17) or even a residual state of neuronal damage.

It has been demonstrated that JCV-genome can be found in brain tissue of patients suffering from other neurological diseases than PML and even in neurologically healthy individuals (18). However, the combined results of our neurological examination, MRI and CSF (19) analyses strongly indicate the diagnosis of PML. This case shows the relevance of close clinical monitoring of apparently immunocompetent patients with PML as a progression of symptoms may be due to the development of a spontaneous, steroid-responsive PML-IRIS.

## Consent for Publication

All authors gave their consent for publication.

## Data Availability

Please contact corresponding author for data requests. The datasets generated and/or analyzed during the current study are available from the corresponding author on reasonable request.

## Ethics Statement

Written informed consent was obtained from the patient and his legal guardian for the participation as well as the publication of the case report and any accompanying images. A copy of the written consent is available upon request from the corresponding author.

## Author Contributions

LK wrote the manuscript and was involved in the diagnostic and therapeutic clinical progress. PR contributed MR-images and legends (Figures 1, 3). RS analyzed PET-data and contributed images for Figure 2. GB contributed to the images for Figure 2 and critically revised the manuscript. MSto analyzed the patient data regarding the immunological aspects and helped in the diagnostic process as well as critically revising the manuscript. MS contributed to the diagnostic and therapeutic progress. FW wrote the manuscript and was responsible for diagnostics and treatment of the patient. All authors read and approved the final manuscript. MS is supported by the Deutsche Forschungsgemeinschaft through the Cluster of Excellence RESIST (EXC 2155).

### Conflict of Interest Statement

The authors declare that the research was conducted in the absence of any commercial or financial relationships that could be construed as a potential conflict of interest.

## Abbreviations

AIDS: acquired immunodeficiency syndrome
CSF: cerebrospinal fluid
HIV: human immunodeficiency virus
IRIS: immune reconstitution inflammatory syndrome
JCV: John-Cunningham-Virus
MRI: magnetic resonance imaging
PCR: polymerase chain reaction
PML: progressive multifocal leukoencephalopathy.
